# Trans-resveratrol reduces visible signs of skin ageing in healthy adult females over 40: an 8-week randomized placebo-controlled trial

**DOI:** 10.3389/fragi.2025.1727244

**Published:** 2025-12-19

**Authors:** Amanda Rao, David Briskey, Georgia Roche, Annie Tremblay, Marcia Da Silva Pinto, Thomas A. Tompkins

**Affiliations:** 1 RDC Clinical, Brisbane, QLD, Australia; 2 School of Human Movement and Nutrition Sciences, University of Queensland, Brisbane, QLD, Australia; 3 Lallemand Bio-Ingredients, Montreal, QC, Canada; 4 Evolva AG, Reinach, Switzerland

**Keywords:** ageing, sebum, skin health, trans-resveratrol, wrinkles

## Abstract

**Introduction:**

This double-blind, randomised, placebo-controlled clinical study evaluated the effects of trans-resveratrol on skin health. To date, and to the best of our knowledge, no study has tested trans-resveratrol as the only active ingredient, orally or topically, for improving skin parameters in humans. Therefore, the aim of this study was to investigate the effects of trans-resveratrol on skin health and visible signs of ageing, when administered orally and/or applied topically to the face.

**Methods:**

Healthy females aged 40 years and older were randomly assigned to one of four groups: placebo oral and topical (P/P Group), trans-resveratrol oral and placebo topical (A/P Group), placebo oral and trans-resveratrol topical (P/A Group), and trans-resveratrol oral and topical (A/A Group). Participants were instructed to take one capsule (75 mg trans-resveratrol) and apply 1 g of cream (1.5% trans-resveratrol) twice daily for 8 weeks. Outcome measures included wrinkle assessment, skin age, temperature, pore size, forehead lines, glabellar lines, Crow’s feet, nasolabial folds, pigmentation, sebum levels, moisture, and elasticity, along with a self-assessment questionnaire, serum trans-resveratrol concentrations, and safety.

**Results:**

Out of 134 participants enrolled, 122 completed the study. Results indicated significantly reduced wrinkle scores in the A/A Group compared to the P/P Group at week 8. All treatment groups showed increased sebum levels, with the active topical groups (P/A and A/A Groups) having significantly higher U-zone sebum at week 8 compared to placebo topical groups (P/P and A/P Groups). No significant differences were found in other skin parameters. Serum trans-resveratrol conjugate levels increased significantly in the A/P and A/A Groups at week 4 and 8. All trial products were shown to be safe with minimal and only mild adverse events recorded in every group.

**Conclusion:**

Oral and topical trans-resveratrol treatment can help improve skin health parameters. When taken orally and applied topically, trans-resveratrol was effective at wrinkle reduction, and when applied topically, it increased sebum levels.

**Clinical Trial Registration:**

identifier ACTRN12621000709842.

## 1 Introduction

Ageing is a natural phenomenon involving intrinsic and extrinsic processes resulting in gradual physiological changes. Visible signs of ageing are particularly apparent on the skin as it is the organ that is most exposed to the outside world (Amaro-Ortiz et al., 2014; Zhang and Duan, 2018; Poljšak et al., 2012; Boo, 2019; Farris et al., 2014). Such visible changes include the accumulation of fine lines, wrinkles, dryness, sagging, translucency, uneven pigmentation and visible pores (Amaro-Ortiz et al., 2014; Zhang and Duan, 2018; Poljšak et al., 2012; Boo, 2019; Farris et al., 2014). While intrinsic ageing is primarily dependent on genetic processes (Boo, 2019), extrinsic factors such as lifestyle choices, sun exposure, and pollution (Boo, 2019) accelerate ageing significantly and account for approximately 97% of ageing events (Zhang and Duan, 2018). As people age, sebum (an oily substance that can protect your skin) production starts to decline due to hormonal changes, especially a decrease in androgen levels (Mosca et al., 2025; Fenske and Lober, 1986). A reduction in sebum can lead to drier skin, which is less elastic and more prone to developing wrinkles and fine lines (Hashizume, 2004; Hou et al., 2022). This is because sebum forms a barrier on the skin’s surface, reducing water loss and keeping the skin hydrated (Schneider and Paus, 2010; Strauss, 2006).

The appearance of the skin, especially wrinkles and fine lines, has a notable social impact by conveying the level of an individual’s attractiveness. Many people desire an outwardly youthful appearance, even as they age (Zhang and Duan, 2018; Boo, 2019). Moreover, disruption of the skin barrier can result in adverse health consequences such as stimulating inflammatory dermatosis and allowing the penetration of microbial pathogens into the skin, both of which may lead to elevated inflammation in the systemic circulation (Wen et al., 2020). As a result, there is heightened investigation into ingredients that improve skin function and visible signs of ageing.

Although extrinsic and intrinsic ageing exhibit somewhat different phenotypes, both processes share a common underlying mechanism: the accumulation of reactive oxygen species (ROS), which modify the activity of lipids, proteins, and DNA (Zhang and Duan, 2018; Poljšak et al., 2012; Boo, 2019; Farris et al., 2014). This is coupled with DNA repair ability decreasing with age (Farris et al., 2014). Natural ingredients are well sought after in this regard because they are better tolerated than synthetic chemicals while having comparable benefits (Wen et al., 2020).

Trans-resveratrol is a polyphenol phytoalexin that plants synthesise in response to external stressors (Farris et al., 2014). It exists in two isomeric forms, cis and trans, where its trans-form is biologically active (Farris et al., 2014). In addition to being found in numerous plant species, trans-resveratrol is present in red wine, grapes, dark chocolate, peanuts and berries (Salehi et al., 2018; Ramírez-Garza et al., 2018; Shaito et al., 2020). Trans-resveratrol’s mechanisms of action include activating the antioxidant Nrf2/ARE, sirtuin 1 (SIRT1), AMPK and MAPK signalling pathways, inhibiting protein kinase D, up-regulating heat-shock protein 27 (HSP27), downregulating caspase 3, preventing the activation of inflammatory transcription factor NFκB, up-regulating antioxidant enzymes, reducing MEK1-p and ERK1/2-P expression, decreasing AP-1 activity, modulating tyrosinase activity, exhibiting oestrogen-like effects and down-regulating numerous other inflammatory pathways (Boo, 2019; Farris et al., 2014; Wen et al., 2020; Ratz-Łyko and Arct, 2019; Baxter, 2008).

Sebum regulation is also a relevant consideration in the context of trans-resveratrol’s dermatological effects. Beyond its antioxidant and anti-inflammatory actions, trans-resveratrol has demonstrated the ability to modulate lipid synthesis within human sebocytes, including down-regulating sterol regulatory element–binding protein-1 (SREBP-1) and key lipogenic enzymes involved in sebum production (Abu-Farha et al., 2016). Resveratrol’s antimicrobial and anti-inflammatory effects against *Cutibacterium acnes* further highlight its activity within the pilosebaceous unit (Sun et al., 2012). Clinical studies of topical resveratrol formulations have also reported reductions in facial sebum levels and improvements in overall skin appearance (Liu and Stauber, 2019). Given that sebum contributes to hydration balance, pore visibility and skin surface texture—parameters frequently altered with age—sebum assessment provides an important biomarker to evaluate the potential cutaneous benefits of trans-resveratrol in an ageing population.

Trans-resveratrol is a generally recognised as safe (GRAS) food ingredient in the USA and an authorized Novel Food in Europe. Its oral and topical usage is safe and well-tolerated in clinical studies up to doses of 1 g (oral) (Salehi et al., 2018; Ramírez-Garza et al., 2018; Shaito et al., 2020) and 3% (topical) (Boo, 2019; Farris et al., 2014; Wen et al., 2020). Though trans-resveratrol’s effects on skin health and ageing are well documented using *in vitro* and *in vivo* models, there are relatively few clinical studies on this topic. Furthermore, to the best of our knowledge, of the clinical trials incorporating trans-resveratrol products for skin health, none have included an only trans-resveratrol treatment group. Clinical trials to date have typically incorporated a mixture of trans-resveratrol with other active ingredients.

Farris et al.’s 2014 study found that a topical blend containing 1% trans-resveratrol, 0.5% baicalin and 1% vitamin E showed significant improvements in fine lines and wrinkles, skin firmness, skin elasticity, skin laxity, hyperpigmentation, radiance and skin roughness in healthy ageing female subjects when applied for 12 weeks. Ultrasound measurements in the periorbital area showed a significant improvement, indicating dermal remodelling. Trans-resveratrol also demonstrated percutaneous absorption and alterations in gene expression of heme oxygenase 1 (HO-1), vascular endothelial growth factor (VEGFA) and collagen 3 (COL3A1) (Farris et al., 2014). Other studies have documented the efficacy of topical trans-resveratrol formulations for treating acne vulgaris (Fabbrocini et al., 2011) and reducing skin wrinkling (Buonocore et al., 2012), facial redness (Ferzli et al., 2013) and systemic oxidative damage (Buonocore et al., 2012). All trans-resveratrol products were well tolerated and were not associated with any adverse effects (Farris et al., 2014; Fabbrocini et al., 2011; Buonocore et al., 2012; Ferzli et al., 2013). However, more clinical studies are needed to ascertain the efficacy of trans-resveratrol, particularly as the only active ingredient, on improving various parameters of skin health.

The aim of this study was to investigate the efficacy of trans-resveratrol compared to a placebo on skin health parameters and visible signs of ageing, when administered orally and/or applied topically to the face in otherwise healthy females aged over 40 years old. It was hypothesised that oral supplementation and topical application of trans-resveratrol to the face would improve facial skin health parameters and ageing, including fine lines, wrinkles, uneven pigmentation, skin elasticity, hydration and visible pores compared to the placebo products.

## 2 Materials and methods

### 2.1 Study design

This was a double-blind, randomised, placebo-controlled clinical study with four groups. The study was conducted in Brisbane, Australia, between September 2021 and November 2022. Potential participants were provided with the participant information sheet, and following screening via a telehealth consultation, acceptable participants gave their written consent to participate in the study and completed baseline measures. Ethics was approved by Bellberry Limited, Human Research Ethics Committee E, approval number 2021-04-341 on 11 June 2021. This study was registered with ANZTCR on 08th June 2021 (ACTRN12621000709842).

### 2.2 Randomisation and groups

Randomisation was performed using Random Allocation Software (Sealedenvelope.com.au) by an individual not involved in the study to ensure both participants and investigators were blinded to the allocation. Once enrolled in the study, participants were randomly allocated to one of the four treatment groups on a 1:1:1:1 ratio. Participants were assigned to one of four groups, each receiving both an oral and a topical product. For each product type, either an active (A) or placebo/vehicle (P) formulation was provided. In all group descriptions throughout, the oral product is listed first, followed by the topical product.

- Group 1 (P/P): placebo oral + placebo/vehicle topical cream
- Group 2 (A/P): active oral (resveratrol capsules) + placebo/vehicle topical cream
- Group 3 (P/A): placebo oral + active topical (resveratrol cream)
- Group 4 (A/A): active oral (resveratrol capsules) + active topical (resveratrol cream)

### 2.3 Investigational products

The active capsules contained 75 mg of trans-resveratrol (Veri-te™, Evolva AG, Reinach, Switzerland) and the placebo capsule consisted of microcrystalline cellulose (MCC). The active treatment cream (Juneo™, Evolva AG, Reinach, Switzerland) was a water based (oil in water emulsion) cream with 1.5% trans-resveratrol. The vehicle/placebo treatment cream included the same ingredients as the active treatment cream with the exclusion of trans-resveratrol. Participants were instructed to take one capsule with water twice daily (morning and evening) and apply 1 g of cream twice daily (morning and evening) after cleaning the face and before applying moisturizer. Placebos were also supplied by Evolva AG (Reinach, Switzerland).

### 2.4 Participants eligibility

One hundred and thirty-four (134) otherwise healthy females were enrolled in this study. Participants were included using the criteria listed in Table 1.

**TABLE 1:** Eligibility and exclusion criteria.

Inclusion criteria
• Females aged 40 years and above • Otherwise, healthy • Agree not to change standard skincare routine • Able to provide informed consent • Agree not to undertake another clinical trial while enrolled in this trial

Exclusion criteria
• Clinically significant medical conditions including, but not limited to, cardiovascular, neurological, psychiatric, renal, gastrointestinal, immunological, endocrine (including uncontrolled diabetes or thyroid disease), or haematological abnormalities that are uncontrolled • Currently taking trans-resveratrol or using trans-resveratrol cream • Cosmetic surgery and procedures, including Botox and other injectables, microdermabrasion, and laser treatments • Medications for acne or other skin conditions including topical retinoids (Rein-A, Retrieve) oral retinoids such as Isotretinoin (Roaccutane), benzoyl peroxide, AHA’s, serum, and chemical peels • Active smokers, nicotine use, alcohol, or drug (prescription or illegal substances) abuse • Chronic past and/or current alcohol use (>14 alcoholic drinks per week) • Known pregnant or lactating woman • Allergies or hypersensitivity to trans-resveratrol • Any condition which in the opinion of the investigator makes the participant unsuitable for inclusion

### 2.5 Outcome measures

Following written consent being given and prior to being provided with study products, baseline measures of all outcomes were collected, and a fasted blood sample taken (Table 2). Prior to the skin assessment using the A-One Smart Pro analyser, participants acclimatized to the clinic’s ambient temperature, removed all make-up using make-up removal wipes and any hair was cleared away from the face.

**TABLE 2:** Primary and secondary evaluation parameters.

Outcome measure	Assessment method
Primary	
• Fine and deep wrinkles	A-one pro^a^
Secondary	
• Assessment of skin age and temperature, pore size, forehead lines, glabellar lines, Crow’s feet, nasolabial folds, pigmentation, sebum [U-zone (cheeks, jawline and chin) and T-zone (forehead, nose, and chin), moisture and elasticity	A-one pro^a^
• Trans-resveratrol concentration (free), trans-resveratrol metabolites (conjugated sulphates and glucuronides)	Serum
• Participant self-assessment questionnaire^b^	Questionnaire
• Adverse reactions	Symptoms report

^a^ The A-one Smart Pro skin analysis system (Bomtech Electronics Co Ltd, Seoul, Korea) uses a high-resolution camera to take photographs of the skin in normal, UV and polarized light and an attached probe measures skin temperature and moisture.

^b^ The self-assessment questionnaire completed at week 8 only.

The A-One Smart Pro captures high-resolution photographs under standardised lighting conditions, including visible light, cross-polarised light and parallel-polarised light. These imaging modes separately evaluate surface texture, subsurface pigmentation, vascular patterns, moisture distribution, and sebum levels. Once images are captured, the integrated software automatically aligns facial landmarks and applies algorithms to quantify a range of skin parameters.

Wrinkle analysis is performed by detecting fine to coarse linear depressions across pre-defined anatomical regions (e.g., forehead lines, glabellar lines, Crow’s feet, nasolabial folds). The software identifies wrinkle length, depth and density. Depth estimation is enhanced through polarised and cross-polarised imaging, which highlights micro-shadowing created by topographical irregularities. The system then compares these measurements against an internal age-matched reference database, generating wrinkle scores that reflect both absolute severity and relative deviation from typical age-related norms.

Other outcome measures—including pore size, pigmentation, elasticity and moisture—are derived through analogous multi-modal imaging and algorithmic interpretation. Pore size is quantified by identifying circular depressions and calculating their average diameter and distribution. Pigmentation is assessed by isolating melanin-rich regions under cross-polarised light, while elasticity and firmness are estimated from micro-distortion patterns captured during image acquisition. Sebum measurements are obtained through parallel-polarised imaging, which enhances the reflectivity of lipid-rich areas on the skin’s surface. Together, these metrics yield a comprehensive, objective profile of each participant’s skin characteristics.

A fasting serum blood sample (6 mL) was taken from the antecubital fossa, allowed to clot for 30 min at room temperature before being centrifuged at 4 °C for 10 min at 1,200 x g (2,400 RPM). Once spun, aliquots of serum were immediately stored at −80 °C until analysis. Analysis of serum trans-resveratrol and its conjugates (sulphates and glucuronides) were conducted using liquid chromatography tandem mass spectrometry (LC-MS-MS) as previously reported (Briskey and Rao, 2020).

Once the participant completed all baseline measurements, they were instructed to take and apply the allocated products for 8 weeks. During the 8-week study period, participants were asked to attend the study site at the midpoint (week 4) and at the end of the study (week 8) to repeat baseline measurements (Table 2). Participants were required to attend the study site in a fasted state during all visits. At the end of the 8-weeks, participation in the study was considered complete. Participants were monitored for compliance by having to return any unused trial products to their final visit, along with a combination of telephone and email communications throughout the study.

### 2.6 Sample size calculation

Sample size was calculated using G*power (v3.1). Statistical parameters were set as an α error probability of 0.05 and powered to 0.80 to detect a 10% difference in wrinkle score between the active and placebo groups. Analysis determined that 29 participants per group were required to complete the study. Allowing for a predicted 20% dropout rate, up to 35 participants per group were to be enrolled (up to 140 participants total).

### 2.7 Data analysis

Analysis was conducted using GraphPad Prism 7.0 and SPSS 22. All results were first tested for normality before any other test was conducted. Based on the distribution of the data, the appropriate statistical test was used as required. Differences between groups were assessed using ANOVA for continuous data or Fisher’s exact test for categorical data. A significant difference between groups was considered at p < 0.05. For between group differences at weeks 4 and 8, data was analysed using change from baseline data.

## 3 Results

**FIGURE 1:**
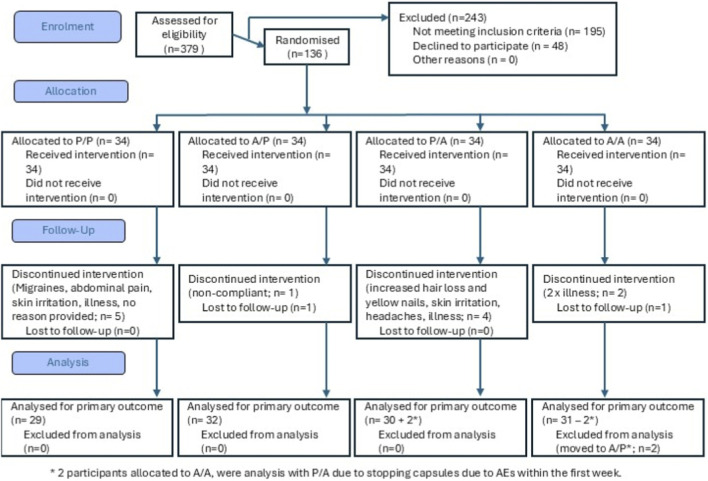
Consort flowchart depicting the progress through the study phases.

Of the 136 participants enrolled, 122 completed the full trial requirements. There was no significant difference in demographics between groups at baseline (Table 3). Of the 14 participants that withdrew, 2 were lost to follow-up, 4 became ill with non-intervention related infections (cold/flu), 1 withdrew for no accountable reason, 1 was withdrawn due to non-compliance and 6 withdrew due to adverse events (1 x increased hair loss and yellow nails, 2 x headaches/migraines, 1 x abdominal pain, 2 x skin irritation) (Figure 1).

**TABLE 3:** Group demographics.

Demographics	P/P group	A/P group	P/A group	A/A group
Number enrolled	34	34	34	34
Number withdrawn	5	2	4	3
Number completed	29	32	30 + 2^a^	31–2^a^
Age (average, SD)	57.6 (8.1)	58.3 (9.0)	59.3 (8.0)	55.8 (8.1)
Height (m)	1.65 (0.07)	1.63 (0.07)	1.63 (0.07)	1.64 (0.06)
Weight (kg)	69.9 (14.7)	69.6 (13.9)	69.3 (13.0)	72.3 (13.7)

^a^ Two participants, allocated to A/A, were analysed with the P/A group due to stopping capsules after experiencing AEs within the first week. Values presented are mean (SD).

Eleven adverse events were reported during the study. Four events were reported in the P/P Group (2 x skin irritation, abdominal pain, and migraines), three events were reported in the A/P Group (headaches, increased hair loss and yellow nails, and skin irritation), two events were reported in the P/A Group (skin irritation and syncope from blood test), and two events were reported in the A/A Group (indigestion and nausea). Of the 11 adverse events reported, all were mild, with six leading to participant withdrawal. Three withdrew from the P/P Group (skin irritation, abdominal pain, and migraines), two from the A/P Group (headaches, increased hair loss, and yellow nails), and one from the P/A Group (skin irritation).

The two adverse events in the A/A group were reported within the first week of dosing, both participants were instructed to stop taking the capsules and continue with the topical. The product allocation remained blinded to study personnel and participants, and, consistent with the blinding code, data from both individuals were subsequently included in the analysis of the P/A group (placebo capsule group). Of the adverse events reported, three events in the P/P Group, one in the A/P Group, two in the P/A Group and two in the A/A Group were considered possibly related to the trial product.

**FIGURE 2:**
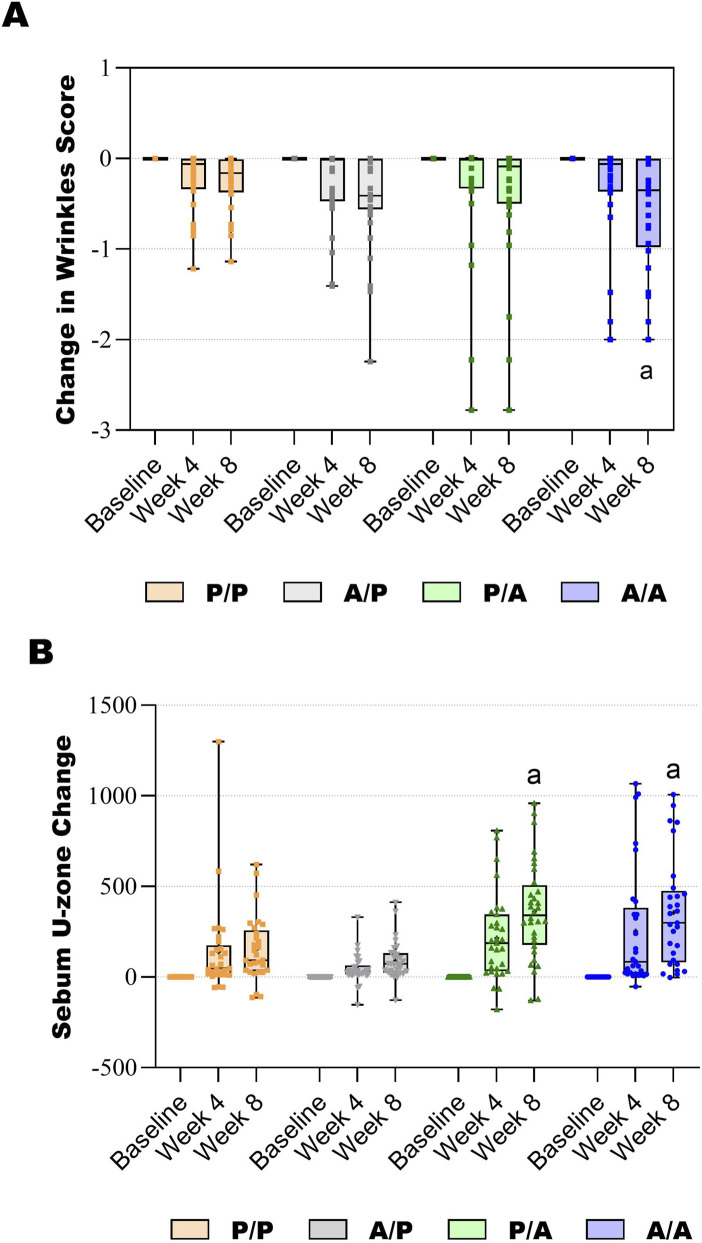
Box and whiskers graphs showing **(A)** the change in wrinkle score over 8 weeks and **(B)** the change in U-zone sebum score over 8 weeks. The lines in the boxes represent the median. a, p < 0.05 vs. the P/P Group.

**FIGURE 3:**
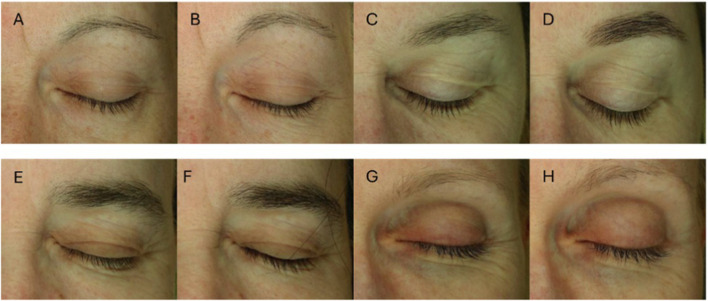
Total wrinkle assessment. Representative facial images illustrating wrinkle appearance at baseline and week 8 for each intervention group. Participants shown were selected because their wrinkle scores were closest to the respective group means reported in Table 4. Wrinkle quantification reflects whole-region periorbital analysis performed by the A-One Smart Pro system rather than changes in a specific focal area. Images show a participant from: P/P Group at baseline **(A)** and week 8 **(B)**. A/P Group at baseline **(C)** and week 8 **(D)**. P/A Group at baseline **(E)** and week 8 **(F)**. A/A Group at baseline **(G)** and week 8 **(H)**.

Wrinkle score was similar between groups at baseline, with the average baseline score of approximately 5, indicating moderate wrinkle appearance. All groups decreased their wrinkle score over the 8 weeks of treatment (p < 0.05), with the A/A Group decreasing significantly more than the P/P Group (p < 0.05) (Table 4; Figures 2A, 3A–H). For secondary wrinkle outcomes, there was no difference between groups at any time point for forehead lines, glabellar lines, crow’s feet, and nasolabial lines (Supplementary Table S1).

**TABLE 4:** Total wrinkles.

​	P/P group	A/P group	P/A group	A/A group
Baseline (score 0–10)	4.90 (1.06)	4.60 (1.10)	5.10 (1.19)	5.04 (1.20)
Week 4 (score 0–10)	4.67 (1.11)	4.43 (0.91)	4.84 (1.04)	4.67 (1.12)
Week 8 (score 0–10)	4.59 (1.10)^a^	4.16 (1.01)^a^	4.70 (1.01)^a^	4.44 (1.03)^a^
Δ week 8	−0.31 (0.32)	−0.44 (0.52)	−0.40 (0.67)	−0.59 (0.64)^b^
Change from baseline (%)	−6.3	−9.6	−7.8	−11.9

Values presented are mean (SD).

^a^ Statistically significant from baseline.

^b^ Statistically significant compared to P/P Group. Significance was set to p < 0.05.

Baseline sebum levels were similar between groups, with measured baseline values indicating low-to-normal sebum levels for people in this age range. Sebum levels significantly increased (p < 0.05) at both week 4 and 8 in all treatment groups. At week 4, the A/A Group increased U-zone sebum significantly more than the A/P Group (p < 0.05). At week 8, the P/A and A/A Groups increased U-zone sebum significantly more than both the P/P and A/P Groups (p < 0.05) (Table 5; Figure 2B).

**TABLE 5:** U-zone sebum score.

​	P/P group	A/P group	P/A group	A/A group
Baseline	438.1 (329.3)	409.2 (257.8)	408.7 (302.7)	327.3 (269.9)
Week 4	577.7 (367.3)^a^	466.8 (240.1)^a^	619.2 (298.2)^a^	581.6 (353.4)^a^
Week 8	590.3 (296.4)^a^	500.0 (245.7)^a^	771.3 (299.8)^a^	683.0 (317.9)^a^
Δ from baseline (0–8)	152.2 (179.4)	90.8 (104.4)	362.6 (270.3)^b^	355.7 (299.3)^b^
Δ from baseline (%)	34.7	22.2	88.7*	108.7

Scored (0-10,000). Values presented are mean (SD).

^a^ Statistically significant from baseline.

^b^ Statistically significant compared to P/P Group. Δ = change, Significance was set to p < 0.05.

**FIGURE 4:**
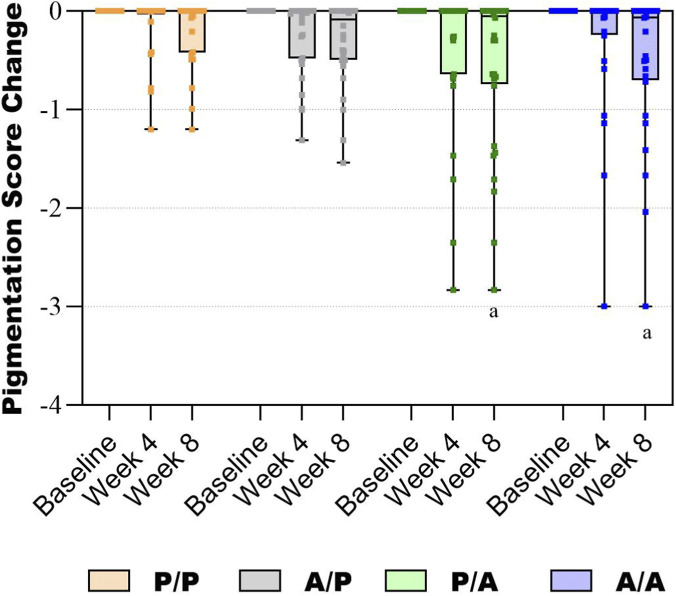
Box and whiskers graph showing change in pigmentation score over 8 weeks. The lines in the boxes represent the median. a, p < 0.05 vs. the P/P Group.

For secondary skin health outcomes, there was no difference between groups at any time point for skin age, skin temperature, pore size, T-zone sebum, moisture, or elasticity (Supplementary Table S2). The change from baseline in pigmentation for the P/A and A/A Groups decreased significantly more than the P/P Group (p < 0.001, Figure 4, Supplementary Table S2).

With respect to serum concentration of trans-resveratrol and its conjugates at week 4, the A/A Group had significantly increased serum trans-resveratrol compared to the P/P and P/A Groups (p < 0.05) and the A/P Group had significantly increased serum trans-resveratrol compared to the P/A Group (p < 0.05). No significant difference was found between any groups at week 8. There was no difference found between groups at baseline for trans-resveratrol sulphates. At week 4 and 8, the A/P and A/A Groups had significantly increased serum trans-resveratrol sulphates compared to the P/P and P/A Groups (p < 0.05). There was no difference found between groups at baseline for trans-resveratrol glucuronides. At week 4, the A/P and A/A Groups had significantly increased serum trans-resveratrol glucuronides compared to the P/P and P/A Groups. At week 8 the A/P and A/A Groups had significantly increased serum trans-resveratrol glucuronides compared to the P/P Group (Supplementary Table S3).

The week 8 self-assessment questionnaire data showed no significant difference between any groups for any individual question (Supplementary Table S4) when considering mean scores.

## 4 Discussion

To date, and to the best of our knowledge, no study has tested trans-resveratrol as the only active ingredient, oral or topical, for improving skin parameters in humans. Therefore, the aim of this study was to investigate the efficacy of trans-resveratrol compared to a placebo on skin health parameters and visible signs of ageing, when administered orally and/or applied topically to the face in otherwise healthy females aged over 40 years old. Of the 134 participants enrolled, 122 completed the 8-week intervention (91% retention). The trial products were well tolerated across the groups with the highest number of adverse events occurring in the P/P group. Events included skin irritation, headaches, gastrointestinal discomfort, and unrelated symptoms such as migraines, syncope during blood collection, increased hair loss and yellowing of the nails. Two events initially reported in the A/A group occurred within the first treatment week and resolved upon stopping the capsule; due to blinded allocation, both participants were subsequently analysed within the placebo-capsule group. Importantly, adverse events occurred across both active and placebo groups, and several were not characteristic of reactions to trans-resveratrol, suggesting that excipients in the cream or capsule bases were more likely contributors than the active compound itself. Future work may therefore benefit from optimisation of the topical vehicle—such as reducing potential irritants (e.g., certain emulsifiers, fragrances, or alcohols), adjusting pH, incorporating barrier-supportive ingredients, or pre-testing the base formulation alone—to further minimise mild irritation while maintaining suitable delivery characteristics.

Overall, the study showed that trans-resveratrol, when applied topically, or consumed orally, was superior to the topical placebo and/or oral placebo for improving aspects of facial skin health. While all groups showed an improvement in wrinkle scores over the 8-week treatment period, the group that used the combination of the Veri-te™ capsule and applied the Juneo™ cream (A/A Group) had the greatest improvement in wrinkle scores. This suggests that the combination of topical and oral trans-resveratrol treatment (A/A Group) was more effective at reducing wrinkle appearance than the dual placebo group (P/P Group) or either trans-resveratrol treatments [oral (A/P) or topical (P/A)] alone. This finding is in keeping with one of the few known studies involving trans-resveratrol and skin health. Buonocore and colleagues (2012), administered a daily capsule for 60 days to 50 healthy male and female participants aged 35–60 years (Buonocore et al., 2012). Each participant received either a capsule contained dried grape extract (containing among other compounds, 8 mg of trans-resveratrol) and dried pomegranate extract or a placebo. After 60 days of supplementation, the active product group (containing trans-resveratrol among other ingredients) reported improved skin moisture and elasticity, while skin roughness and depth of wrinkles had reduced compared to the placebo (Buonocore et al., 2012). However, as this study involved potentially multiple active compounds, it is hard to determine what effects were due to the addition of trans-resveratrol. The amount of trans-resveratrol used in the study was also less than that of the current study (8 mg vs. 75 mg respectively), making comparison difficult.

A study conducted by Farris and colleagues (2014) applied a formulation containing trans-resveratrol of unknown concentration as well as baicalin and vitamin E for 12-weeks to 55 female subjects aged 40–60 years. Following treatment, improvements in fine lines, wrinkles, firmness, elasticity, skin laxity, skin tone, hyperpigmentation, radiance and tactile roughness was noted as early as week 4 (Farris et al., 2014). The improvement in wrinkles shown by Farris et al. is similar to that of the current study for the group consuming and applying trans-resveratrol and offers supporting evidence for the effectiveness of trans-resveratrol for skin health. However, while the results by Farris et al. are positive, it is difficult to conclude the specific effect of trans-resveratrol without knowing the concentrations of each active ingredient, and without the use of a comparator/placebo group.

Studies involving trans-resveratrol on skin health in humans with respect to ageing are limited. However, animal models have shown promising results and indicated potential mechanisms of action. A study by Kim and colleagues (2019) administered trans-resveratrol to mice and found trans-resveratrol protected against UVB-induced epidermal thickening and wrinkle formation. It was also shown that trans-resveratrol induced Nrf2-dependent antioxidant enzymes and inhibited metalloproteinases. Therefore, it was deemed probable that trans-resveratrol protected against the effects of UVB through activation of the Nrf2/HO-1 signalling pathway (Kim et al., 2019). While the current study analysed serum for trans-resveratrol concentration, it would be useful for future studies to also analyse potential signalling pathways, such as the Nrf2/HO-1 pathway.

One possible mechanism for the improvement in wrinkles scores in the trans-resveratrol groups presented in this paper, may be due to the reported improvement in sebum in the U-zone. Overall, the U-zone sebum of the four groups in this study presented average values that would class them as having low-normal sebum for their age groups. However, the reported U-zone sebum scores are considered significantly below normal when compared to younger cohorts (under 40 years old), whose normative range starts above the upper limit of the normal range for those over 40 and tends to be associated with ‘healthier'-looking skin. Therefore, increased U-zone sebum production for those over 40 years old, may indicate skin function to be regenerating and providing better protection due to sebum production reducing with age (Fenske and Lober, 1986; Ferrari et al., 2000) that can lead to drier skin and the development of wrinkles (Hashizume, 2004; Hou et al., 2022).

While all groups in this study increased their U-zone sebum levels, the two groups applying topical trans-resveratrol (P/A and A/A Groups), regardless of oral supplementation, significantly increase U-zone sebum 2-4 times that of the active placebo topical groups (P/P and A/P Groups). This suggests that trans-resveratrol topical application, may be more effective at protecting the skin compared to a standard moisturising cream. The beneficial effect of increased sebum production is through sebum’s role in maintaining skin health by forming a barrier on the skin’s surface, reducing water loss and keeping the skin hydrated (Schneider and Paus, 2010; Strauss, 2006). Sebum also contains compounds that have antimicrobial and pro- and anti-inflammatory functions, helping to regulate the activity of xenobiotics and can both repair and protect the skin (Zouboulis, 2004). Sebum therefore acts to shield the skin against damaging factors such as pollution, wind, and UV.

The findings of this study are somewhat in contrast to a lot of the published work involving trans-resveratrol and sebum/sebocytes. To date, studies involving trans-resveratrol for skin health have focused primarily on the reduction of sebum for antimicrobial activity and reducing acne. Studies involving skin health and trans-resveratrol have also been conducted mostly using cell or animal models. Fabbrocini and colleagues (2011) conducted a human *in vivo* study on 20 healthy participants aged 18–23 years with facial acne vulgaris. Participants applied a 1% trans-resveratrol formulation to one side of their face, and the vehicle to the other for 60-days. The trans-resveratrol treated side showed a significant reduction in acne compared to the vehicle side (53.75% vs. 6.1% reduction) (Fabbrocini et al., 2011). However, skin parameters such as sebum, were not analysed in this study and therefore it cannot be directly compared to the present study.

Of the *in vitro* studies conducted, trans-resveratrol has been proposed to limit sebum production by limiting the growth and proliferation of sebocytes. This was attributed to the inactivation of extracellular signal-regulated protein kinase (ERK), Akt, peroxisome proliferator-activated receptor (PPAR)-γ and cyclin D1 synthesis, while stimulating p21WAF1/CIP1 (p21) and p27KIP1 (p27) synthesis (Kim et al., 2015). It may therefore be possible that in the event of low sebum production, trans-resveratrol may act in the opposite way to help stimulate sebum production. It is however difficult to translate *in vitro* results to *in vivo* settings due to the difficulty in comparing the doses. The *in vitro* study by Kim and colleagues applied trans-resveratrol at 10 μg/mL to the cells. Our study involved the daily consumption of 150 mg of trans-resveratrol or the application of 2 g of cream (30 mg of trans-resveratrol) to the face. Even if 100% of the consumed trans-resveratrol dose was absorbed, this would equate to approximately 2 mg/kg body weight, or 2 μg/g body weight. The other factor to be considered when assessing trans-resveratrol *in vitro* studies is the form of trans-resveratrol. As previously presented (Briskey and Rao, 2020), and seen here (Table 4), trans-resveratrol is almost completely conjugated within the intestinal tract. Therefore, the effect of trans-resveratrol applied to cells may be different to the effect when consumed and conjugated. Therefore, the effects of trans-resveratrol sulphates and trans-resveratrol glucuronides should be considered with future *in vitro* studies. Similarly, the impact of the intestinal and skin microbiota composition on the transformation of the trans-resveratrol to known bioactive metabolites, such as Lunularin, dihydroresveratrol and 3,4′-O-dihydrocy-trans-stilbene, should be evaluated.

Several factors need to be taken into consideration for the interpretation of the results. As seasonal conditions can affect skin physiology, environmental variation could contribute to the outcomes observed. However, the present study was conducted in Brisbane, Australia, where shifts in temperature and humidity across seasons are comparatively moderate and therefore less likely to produce pronounced changes in sebum secretion, hydration, or wrinkle visibility. All skin assessments were performed in a climate-controlled clinic under standardised ambient conditions, with participants acclimatising prior to imaging, thereby minimising acute environmental influences. Data collection and randomisation occurred continuously throughout the study period, ensuring seasonal distribution across all treatment arms. Therefore, seasonal variability is unlikely to account for the differences observed between our results and those reported in earlier studies.

A known determinant of sebum secretion is circulating androgen concentration, which declines with age and contributes to reduced baseline sebum output in older adults (Rasetto et al., 2004). Androgens such as testosterone and dihydrotestosterone regulate sebaceous gland activity and lipid synthesis, and decreases in these hormones are closely associated with lower sebum production (Atwood et al., 2014; McKee et al., 2018). Although serum was collected for quantifying trans-resveratrol and its metabolites, androgen profiles (e.g., testosterone, DHEA-S) were not analysed in the present study. This represents an additional limitation, as incorporating endocrine markers would have strengthened mechanistic interpretation and enabled a clearer understanding of whether changes in sebum production may have been hormonally mediated or partially “rescued” in response to trans-resveratrol. Future studies would benefit from analysing androgen status alongside skin-imaging outcomes to clarify potential endocrine contributions to sebum modulation in ageing populations.

Collagen remodelling represents another pathway that may underlie the improvements observed in this study but was not assessed. Type I collagen constitutes roughly 80% of dermal collagen, and its gradual decline with age contributes to wrinkle formation and reduced skin elasticity (Sachana et al., 2001; Kumarihamy et al., 2020). Resveratrol has been shown in experimental models to enhance procollagen I synthesis and suppress matrix metalloproteinases (MMP-1, MMP-3, MMP-9), thereby limiting collagen degradation and supporting extracellular matrix integrity (Wang et al., 2014). As collagen biomarkers or imaging-based assessments were not collected, we cannot determine whether the wrinkle reductions seen in the trans-resveratrol groups were accompanied by measurable changes in collagen turnover. Future studies incorporating collagen-related biomarkers—such as procollagen peptides or MMP activity—would help clarify whether trans-resveratrol exerts direct effects on dermal collagen metabolism *in vivo*.

A further limitation of this study is that the study did not assess serum markers of cellular senescence, which reduces the ability to draw conclusions regarding the molecular ageing pathways potentially affected by trans-resveratrol. Given that skin ageing is influenced by both local and systemic processes, future trials incorporating senescence-related biomarkers may help delineate whether the observed improvements reflect localised skin effects or broader biological modulation.

It is difficult to compare the results of our study to other studies due to the differences in designs (i.e., dose, product ingredients, duration, measurement tools). The primary difference with our study is that other studies used a combination of trans-resveratrol with other active ingredients. *In vitro* studies suggest that trans-resveratrol may reduce sebum by downregulating sebocyte activity, and limited *in vivo* data indicate a reduction in acne, which is typically associated with excess sebum. In contrast, our study found that trans-resveratrol increased sebum production. Trans-resveratrol may therefore potentially act as a sebum regulating compound and act to prevent acne in early years via reduction of sebum production when it is high but protect the skin through increased sebum production in later years when sebum production is low. This effect may help with wrinkle prevention and other signs of ageing in the later years. Exploring the sebum regulation potential of trans-resveratrol in follow-up studies would help better understand its effect and help with the development of better dermatology and skin care products. Future *in vivo* studies including pathology analyses would help identify pathways altered by trans-resveratrol. It may also be useful to include a control group composed of healthy younger women in future studies investigating skin age effects. Further studies may also benefit from incorporating a longer treatment period.

## Data Availability

The raw data supporting the conclusions of this article will be made available by the authors, without undue reservation.
