# Supplementary material for: Trans-resveratrol reduces visible signs of skin ageing in healthy adult females over 40: an 8-week randomized placebo-controlled trial
Source: Front Aging. 2025 Dec 19;6:1727244. doi: 10.3389/fragi.2025.1727244 (PMC12757695; doi:10.3389/fragi.2025.1727244)
Supplement: Supplementary file 1 [file Table1.docx]

**Supplemental Data**

**Table S1**: Secondary wrinkle outcome measures

|  |  | Forehead Lines | Glabellar Lines | Crow's Feet | Nasolabial Folds |
| --- | --- | --- | --- | --- | --- |
|  |  |  |  |  |  |
| P/P Group | Baseline | 3.00 (1.42) | 4.35 (1.27) | 3.76 (0.98) | 3.49 (1.36) |
|  | Week 4 | 3.28 (1.31) | 4.68 (1.70) | 3.69 (0.97) | 3.43 (1.31) |
|  | Week 8 | 3.48 (1.30) | 4.56 (1.41) | 3.86 (0.88) | 3.38 (1.30) |
|  | Change (0-8) | 0.48 (1.32) | 0.21 (0.76) | 0.10 (0.56) | -0.11 (0.88) |
| A/P Group | Baseline | 3.09 (1.10) | 4.42 (1.21) | 3.55 (0.93) | 3.04 (0.88) |
|  | Week 4 | 3.29 (1.15) | 4.51 (1.04) | 3.99 (1.04) | 3.13 (0.94) |
|  | Week 8 | 3.09 (0.97) | 4.26 (1.15) | 3.68 (1.19) | 2.94 (0.78) |
|  | Change (0-8) | 0.00 (0.78) | -0.16 (0.81) | 0.12 (0.89) | -0.09 (0.55) |
| P/A Group | Baseline | 3.64 (1.52) | 4.77 (1.07) | 3.78 (0.70) | 3.28 (1.08) |
|  | Week 4 | 3.93 (1.82) | 4.86 (1.31) | 4.09 (0.97) | 3.28 (1.16) |
|  | Week 8 | 3.74 (1.47) | 4.88 (1.19) | 4.09 (0.91) | 3.41 (1.22) |
|  | Change (0-8) | 0.10 (1.55) | 0.11 (0.95) | 0.31 (0.73) | 0.12 (0.80) |
| A/A Group | Baseline | 3.02 (0.94) | 4.65 (1.13) | 3.50 (0.90) | 3.16 (1.28) |
|  | Week 4 | 3.31 (0.77) | 4.75 (1.08) | 3.69 (0.91) | 2.96 (1.18) |
|  | Week 8 | 3.01 (0.66) | 4.56 (1.13) | 3.65 (0.86) | 2.89 (0.98) |
|  | Change (0-8) | -0.01 (0.65) | -0.09 (0.67) | 0.15 (0.72) | -0.27 (0.90) |
| p-value^a^ |  | 0.35 | 0.23 | 0.64 | 0.34 |

^a^ p-value represents the overall Welch’s ANOVA test value.

**Table S2**: Secondary skin health outcome measures

|  |  | Skin age | Temperature | Pore size | Pigmentation | Sebum T-Zone | Moisture Level | Elasticity |
| --- | --- | --- | --- | --- | --- | --- | --- | --- |
| P/P Group | Baseline | 59.79 (8.4) | 20.38 (1.27) | 6.56 (0.46) | 5.05 (1.05) | 433.52 (449.29) | 29.59 (3.66) | 40.38 (20.72) |
|  | Week 4 | 59.24 (8.56) | 20.62 (1.37) | 6.43 (0.44) | 4.90 (0.99) | 557.86 (363.3) | 30.97 (4.37) | 54.38 (19.34) |
|  | Week 8 | 58.83 (8.86) | 20.17 (1.51) | 6.4 (0.49) | 4.85 (0.97) | 643 (333.3) | 31.66 (4.79) | 60.76 (16.89) |
|  | Change (0-8) | -0.97 (1.21) | -0.21 (1.57) | -0.16 (0.18) | -0.20 (0.34) | 209.48 (448.95) | 1.79 (2.38) | 20.38 (18.78) |
| A/P Group | Baseline | 60.56 (9.03) | 20.41 (1.74) | 6.54 (0.36) | 5.22 (1.27) | 388.03 (351.85) | 28.91 (3.52) | 37.63 (24.11) |
|  | Week 4 | 60.23 (8.9) | 20.37 (1.73) | 6.45 (0.34) | 5.01 (1.29) | 474.23 (336.62) | 29.57 (3.75) | 47.87 (20.62) |
|  | Week 8 | 59.66 (9.11) | 20.34 (2.09) | 6.4 (0.35) | 4.91 (1.23) | 567.72 (367.87) | 30.75 (4.25) | 58.06 (15.59) |
|  | Change (0-8) | -0.91 (0.82) | -0.06 (2.35) | -0.14 (0.16) | -0.31 (0.41) | 179.69 (259.53) | 1.84 (3.45) | 20.44 (19.24) |
| P/A Group | Baseline | 61.45 (7.77) | 19.84 (1.87) | 6.55 (0.36) | 5.45 (1.11) | 522.63 (809.22) | 29.22 (3.54) | 36.72 (25.15) |
|  | Week 4 | 60.83 (7.76) | 19.68 (1.96) | 6.43 (0.34) | 5.09 (0.9) | 574.13 (369.05) | 31.16 (4.61) | 43.87 (24.1) |
|  | Week 8 | 60.16 (7.89) | 20.16 (2.24) | 6.36 (0.32) | 4.91 (0.95) | 748.69 (360.19) | 32.16 (5.01) | 51.81 (24.24) |
|  | Change (0-8) | -1.25 (0.84) | 0.31 (2.02) | -0.19 (0.21) | -0.54 (0.78)^a^ | 226.06 (727.83) | 2.94 (3.21) | 15.09 (18.43) |
| A/A Group | Baseline | 58.28 (8.29) | 20.69 (1.54) | 6.53 (0.35) | 5.21 (1.25) | 401.66 (651.4) | 28.97 (2.98) | 35.55 (19.44) |
|  | Week 4 | 57.41 (8.36) | 20.38 (1.37) | 6.41 (0.33) | 4.90 (1.25) | 649.45 (464.02) | 29.93 (3.31) | 44.38 (19.47) |
|  | Week 8 | 56.93 (8.47) | 19.97 (1.59) | 6.35 (0.33) | 4.70 (1.10) | 744.21 (415.47) | 31.21 (4.38) | 47.59 (19.28) |
|  | Change (0-8) | -1.34 (0.81) | -0.72 (2.1) | -0.19 (0.18) | -0.50 (0.75) | 342.55 (701.53) | 2.24 (2.71) | 12.03 (12.85) |
| p-value^b^ |  | 0.15 | 0.29 | 0.61 | 0.09 | 0.70 | 0.43 | 0.12 |

^a^ significant compared to the P/P Group, ^b^ p-value represents the overall Welch’s ANOVA test value. Significance was set to p<0.05.

**Table S3**: Serum concentration of trans-resveratrol and its conjugates.

|  |  | Trans-resveratrol (ng/mL) | Trans-resveratrol Sulphates  (ng/mL) | Trans-resveratrol Glucuronides  (ng/mL) |
| --- | --- | --- | --- | --- |
| P/P Group | Baseline | 0 (0) | 58.3 (163.4) | 8.1 (10.5) |
|  | Week 4 | 0 (0) | 52 (110) | 11.6 (17.5) |
|  | Week 8 | 0 (0) | 36.8 (86.3) | 11.5 (25.1) |
|  | Change (0-8) | 0 (0) | -21.6 (128.7) | 3.4 (28.9) |
| A/P Group | Baseline | 0 (0) | 76.4 (238.2) | 6.5 (10.1) |
|  | Week 4 | 7.2 (13.1)^c^ | 4158.1 (5622)^b,c^ | 102.4 (206.6)^b,c^ |
|  | Week 8 | 4.6 (14) | 3384.9 (4955.4)^b,c^ | 123.9 (231.1)^b^ |
|  | Change (0-8) | 4.6 (14) | 3087.3 (4882.9) | 117.3 (232.7) |
| P/A Group | Baseline | 0 (0) | 61.3 (140.5) | 6.9 (15.3) |
|  | Week 4 | 0 (0) | 125.6 (215.3) | 6.6 (17) |
|  | Week 8 | 0 (0) | 59.7 (114.3) | 19.9 (42.7) |
|  | Change (0-8) | 0 (0) | 25.4 (131.9) | 12.3 (47.6) |
| A/A Group | Baseline | 0 (0) | 79.5 (328.3) | 7 (12.3) |
|  | Week 4 | 8.7 (15.1)^b,c^ | 5877.2 (13000.8)^b,c^ | 106.6 (177.2)^b,c^ |
|  | Week 8 | 0.2 (0.7) | 3957.7 (5234.5)^b,c^ | 89.8 (123)^b^ |
|  | Change (0-8) | 0.2 (0.7) | 3947.9 (5236.5) | 82.5 (125.8) |
| p-value^a^ |  | 0.14 | <0.0001 | 0.0012 |

^a^ p-value represents the overall Kruskal-Wallis ANOVA test value. ^b^ Significant compared to the P/P Group. ^c^ Significant compared to the Group P/A. Significance was set to p<0.05.

**Table S4**: Week 8 self-assessment questionnaire data

|  | P/P Group 1 | A/P Group | P/A Group | A/A Group | p-value^a^ |
| --- | --- | --- | --- | --- | --- |
| I am satisfied with the overall results this product provides | 18/10 (3.68) | 22/7 (3.79) | 22/10 (3.69) | 24/7 (3.97) | 0.66 |
| My lines and wrinkles are less visible | 10/18 (3.25) | 11/18 (3.28) | 15/17 (3.34) | 15/16 (3.52) | 0.70 |
| My face appears more firm | 13/15 (3.43) | 16/13 (3.48) | 16/16 (3.38) | 18/13 (3.68) | 0.82 |
| My skin tone has become more even | 12/16 (3.18) | 10/19 (3.24) | 13/19 (3.22) | 17/14 (3.61) | 0.46 |
| My skin brightness has increased | 9/19 (3.18) | 12/17 (3.34) | 11/21 (3.22) | 17/14 (3.61) | 0.28 |
| My skin texture has improved | 17/11 (3.54) | 20/9 (3.76) | 18/14 (3.44) | 22/9 (3.87) | 0.60 |
| My skin looks refreshed | 14/14 (3.46) | 16/13 (3.55) | 17/15 (3.44) | 20/11 (3.68) | 0.71 |
| My skin feels more resilient | 8/20 (3.07) | 11/18 (3.31) | 15/17 (3.31) | 19/12 (3.77) | 0.07 |
| My skin clarity has improved | 12/16 (3.29) | 7/22 (3.24) | 14/18 (3.28) | 20/11 (3.77) | 0.02 |
| My face feels rejuvenated | 11/17 (3.21) | 9/20 (3.21) | 14/18 (3.25) | 18/13 (3.65) | 0.20 |
| My skin has a youthful glow/radiance | 11/17 (3.21) | 9/20 (3.1) | 13/19 (3.25) | 13/18 (3.32) | 0.82 |
| My skin feels more firm | 9/19 (3.18) | 16/13 (3.52) | 12/20 (3.19) | 18/13 (3.65) | 0.12 |
| My skin looks and feels more hydrated | 20/8 (3.86) | 20/9 (3.66) | 18/14 (3.5) | 24/7 (3.94) | 0.35 |
| My skin looks more healthy | 15/13 (3.46) | 14/15 (3.45) | 15/17 (3.31) | 20/11 (3.74) | 0.49 |
| My skin feels more balanced | 12/16 (3.36) | 8/21 (3.21) | 13/19 (3.25) | 15/16 (3.52) | 0.40 |
| My pores appear less visible | 8/20 (3.14) | 9/20 (3.28) | 14/18 (3.34) | 9/22 (3.19) | 0.56 |
| My skin feels soothed | 15/13 (3.57) | 12/17 (3.38) | 20/12 (3.59) | 24/7 (3.87) | 0.03 |

Values presented are the sum of people who “strongly agree and agree” / “neutral, disagree and strongly disagree” (mean score).

^a^ p-value represents the overall Fisher’s exact test value. Significance was set to p<0.05.
